# Therapeutic potential of isochlorogenic acid A from *Taraxacum officinale* in improving immune response and enhancing the efficacy of PD-1/PD-L1 blockade in triple-negative breast cancer

**DOI:** 10.3389/fimmu.2025.1529710

**Published:** 2025-03-05

**Authors:** Tangyi Wang, Jingwei Sun, Li Wang, Yuxin Lin, Zhijing Wu, Qiangqiang Jia, Shoude Zhang, Juan An, Xueman Ma, Qiong Wu, Zhanhai Su, Haiyan Wang

**Affiliations:** ^1^ Department of Basic Medical Sciences, Qinghai University Medical College, Xining, Qinghai, China; ^2^ Department of Medical Laboratory, Qinghai Provincial People’s Hospital, Xining, Qinghai, China; ^3^ State Key Laboratory of Plateau Ecology and Agriculture, Qinghai University, Xining, Qinghai, China; ^4^ Research Center for High Altitude Medicine, Qinghai University, Xining, Qinghai, China; ^5^ Key Laboratory of the Ministry of High Altitude Medicine, Qinghai University, Xining, Qinghai, China

**Keywords:** immune checkpoint blockade, *Taraxacum officinale*, PD-1/PD-L1 inhibitor 2, tumor microenvironments, triple-negative breast cancer

## Abstract

**Introduction:**

*Taraxacum officinale*, a traditional medicinal herb, has garnered significant attention for its potential role in the prevention and treatment of breast cancer. Although clinical recognition of its efficacy has gradually increased, research has shown that *Taraxacum officinale* contains a variety of chemical components, including triterpenes, carbohydrates, flavonoids, phenolic acids, sesquiterpenes, coumarins, fatty acids, and organic acids. However, the pharmacological mechanisms underlying *Taraxacum officinale*’s effects and the identification of its key bioactive components warrant further investigation.

**Methods:**

Flow cytometry was utilized to investigate the effects of Taraxacum officinale extract (TOE) in combination with PD-1/PD-L1 inhibitor 2 on the immune microenvironment of triple-negative breast cancer (TNBC). Active compounds and their potential targets were identified through an integrative approach involving GeneCards, OMIM, and DisGeNET databases, as well as UPLC-Q-Orbitrap MS analysis. Gene Ontology (GO) and Kyoto Encyclopedia of Genes and Genomes (KEGG) pathway enrichment analyses were conducted, followed by molecular docking to explore compound-target interactions. The anti-proliferative effects of isochlorogenic acid A (ICGA-A) and chicoric acid (CRA) on MDA-MB-231 and 4T1 cells were evaluated using the CCK-8 assay. *In vivo* validation was performed using a 4T1 murine model and flow cytometry.

**Results:**

TOE and its active constituents, ICGA-A and CRA, demonstrate potential in augmenting PD-1 blockade therapy for TNBC. This study investigated the combination of ICGA-A and PD-1/PD-L1 inhibitor 2, which significantly enhanced the infiltration of macrophages and CD8+ T cells into tumors in murine models, while concurrently reducing the population of exhausted T cells. Furthermore, CRA notably increased the frequency of CD8+ T cells. Both ICGA-A and CRA therapies were also found to suppress tumor proliferation by inhibiting the FAK/PI3K/AKT/mTOR signaling pathway. These findings highlight the potential of ICGA-A and CRA as effective adjuvants to improve the therapeutic efficacy of PD-1 inhibitor-based immunotherapy in TNBC.

**Discussion:**

ICGA-A and CRA, bioactive compounds from *Taraxacum officinale*, exhibit significant antitumor activity in TNBC by targeting the FAK/PI3K/AKT/mTOR pathway, a critical regulator of cancer progression. Their ability to modulate the tumor immune microenvironment highlights their potential as immune modulators that enhance the efficacy of immunotherapy. These findings suggest that ICGA-A and CRA could serve as promising adjuncts in TNBC treatment, offering a novel strategy to overcome challenges such as therapeutic resistance and limited treatment options. Further investigation is warranted to explore their synergistic effects with immunotherapies in improving TNBC outcomes.

## 1 Introduction

Immunotherapy has revolutionized cancer treatment, particularly through the use of immune checkpoint inhibitors (ICIs), which have shown remarkable efficacy in certain cancers. However, the low response rates to ICIs remain a significant challenge, especially in breast cancer, where the response rate is approximately 20% (1–3). As a result, much research has focused on combination therapies to enhance the efficacy of ICIs. In our previous study, using high-throughput screening based on HTS^2^ (high-throughput sequencing-based high-throughput screening) of over 2,000 compounds, we identified an aurora kinase inhibitor that, when combined with a PD-1 inhibitor, effectively inhibited the growth of TNBC by modulating the JAK1/STAT3 pathway and promoting CD8+ T cell infiltration in the tumor microenvironment (4). However, traditional medicines, particularly herbal medicines from Chinese and Tibetan sources, offer a reservoir of low-toxicity, bioactive compounds that may present novel therapeutic combinations for ICIs (5).

One such promising candidate is *Taraxacum officinale* extract (TOE), commonly known as dandelion. This herb, widely distributed across regions such as Qinghai, Gansu, Liaoning, and Xinjiang, has been historically utilized in traditional Chinese medicine for treating breast abscesses in women due to its heat-clearing, detoxifying, and anti-swelling properties, as documented in ancient texts like the Tang Bencao (6–8). Recent studies have demonstrated that *Taraxacum officinale* exhibits anti-tumor activity against various cancers, including liver, colorectal, prostate, and leukemia (9–12). The anti-cancer mechanisms of *Taraxacum officinale* extend beyond the inhibition of the PI3K/Akt pathway and activation of p53-mediated apoptosis. It also modulates immune-related pathways, such as IL-10/STAT3 and PD-1/PD-L1, suggesting its potential in reshaping the tumor immune microenvironment (13–15).

## 2 Materials and methods

### 2.1 Extraction and preparation of *Taraxacum officinale*

*Taraxacum officinale* (Product Standard Number: GHT1091) was purchased from Liaoning Senkangyuan Ecological Agriculture Co., Ltd., and authenticated by Wang Kun. The dried whole plant was subjected to reflux extraction with 75% ethanol at 60°C for three cycles, each lasting 3 hours. The combined extracts were filtered and concentrated under reduced pressure to obtain the crude extract. The crude extract was then further purified using D-101 macroporous resin column chromatography. Initially, the column was washed with water to remove hydrophilic impurities such as sugars, followed by elution with 100% ethanol to collect the active compounds. The ethanol fraction was subsequently evaporated under vacuum and spray-dried to obtain the final TOE. For *in vitro* studies, TOE was dissolved in 50% DMSO to prepare a stock solution, which was then diluted with cell culture medium. The final DMSO concentration was maintained at 0.5% to minimize solvent-induced cytotoxicity.

### 2.2 Pretreatment of samples for UPLC-Q-Orbitrap MS

0.50 g of TOE was taken and mixed with 10 ml of solvent (methanol, acetonitrile, and water = 2:2:1), centrifuged at 13000 rpm for 5 min, and filtered through a 0.22 µm membrane filter.

### 2.3 UPLC-Q-Orbitrap MS analysis

A heat electro-spray ionization (HESI) was paired with a Q-Orbitrap MS (Thermo Fisher Scientific) in the MS analysis. The main parameters included the scan range, 100.0 to 1300.0 m/z; spray voltage, 2.80 KV (negative mode); capillary temperature, 300°C; aux gas heater temperature, 300°C; sheath gas flow rate, 40 arbitrary units; aux gas flow rate, 10 arbitrary units; maximum injection time, 50 ms; automatic gain control target, 1.0 e6. Molecular weights [M-H]- and [M-H]+ were used for qualitative analysis. UPLC separation was carried out on an AccucoreaQ C18 column (150 mm×2.1 mm, 2.6 μm, Thermo Fisher Scientific) with a flow rate of 0.3 μL/min. The optimized mobile phase was a mixture of water with 0.9% acetic acid (A) and methanol (B). For identification samples, the gradient was operated as follows: 0-9 min, 20%-100% B; 9-10 min, held at 100% B; 10-11 min, 100%-20% B; and 11-14 min, held at 20% B. The injection volume was 3 μL, and the temperature of the column was 30°C.

### 2.4 Cell culture

The 4T1 and MDA-MB-231 cell lines were obtained from the Procell Life Science & Technology Co., Ltd. MDA-MB-231 cells were cultured in Dulbecco’s modified Eagle’s medium (DMEM) supplemented with 10% fetal bovine serum (Procell) and 1% penicillin/streptomycin (Procell). 4T1 cells were cultured in Roswell Park Memorial Institute 1640 (RPMI-1640) supplemented with 10% fetal bovine serum (Procell) and 1% penicillin/streptomycin (Procell). Cells were cultured at 37°C with a 5% CO2 atmosphere.

### 2.5 CCK8 assays

CCK-8 assays were performed using the CCK-8 kit (Elabscience, China) according to the manufacturer’s instructions. Briefly, MDA-MB-231 or 4T1 cells were seeded at a density of 2.0×10^5 cells/mL in 96-well plates and incubated with culture medium at 37°C and 5% CO2 for 24 hours. MDA-MB-231 and 4T1 cells were then treated with a series of concentrations of TOE for 24 hours (24, 48, 72 hours). CCK-8 assay results indicated that TOE exhibited the most significant inhibitory effect on the viability of TNBC cells at 24 hours (**Supplementary Figure S1**). To maintain consistency, the 24-hour time point was also chosen for assessing the effects of ICGA-A and CRA on cell viability. At the end of the treatment, the culture medium was removed, and 20 µL of CCK-8 reagent was added to each well. The plates were incubated for an additional 1 hour, and the absorbance of each well was measured at 450 nm.

### 2.6 Transwell assays

Cell migration and invasion were assessed using Transwell assays. For the migration assay, approximately 2×10^4 cells were seeded into the upper chamber of the Transwell, which had been treated with TOE, ICGA-A, and CRA, using serum-free medium, while 750 μL of medium was added to the lower chamber. For the invasion assay, 30 µg of Matrigel Matrix (Corning) was added to the upper chamber and incubated for 1 hour at 37°C before seeding the cells. After 20 hours of incubation at 37°C with 5% CO_2_, the medium was removed and the cells were washed twice with PBS. The cells were fixed in paraformaldehyde for 15 minutes and then washed twice with PBS. The chambers were stained with 0.1% crystal violet for 10 minutes, and five fields of view were selected, photographed, and counted under a microscope to evaluate migration and invasion.

### 2.7 Annexin V apoptotic assay

Cells were seeded at a density of 2.5 × 10^5 cells per well in six-well plates and allowed to adhere overnight. The following day, the cells were treated with ICGA-A and CRA for 24 hours. After treatment, both adherent and floating cells were collected by trypsinization (using trypsin without EDTA, Procell) and centrifuged at 300 × g for 5 minutes at 4°C. The cell pellets were then washed twice with cold PBS and resuspended in 100 µL of 1× Annexin V binding buffer. Subsequently, 5 µL of Annexin V-FITC and 5 µL of propidium iodide (PI) were added to each sample, followed by incubation in the dark at room temperature for 15 minutes. After staining, 400 µL of 1× binding buffer was added to each sample. Apoptosis was analyzed using a flow cytometer (Beckman Coulter, China) within 1 hour. Apoptotic populations were identified based on fluorescence signals, with Annexin V^+^/PI^-^ indicating early apoptosis and Annexin V^+^/PI^-^ indicating late apoptosis. All antibodies and buffers were provided by Elabscience Biotechnology Co., Ltd., with antibodies diluted at a 1:2 ratio. Data analysis was performed using FlowJo software.

### 2.8 Animal model construction

To establish the tumor model, 10 g of tribromoethanol (Sigma) was dissolved in 10 mL of tert-pentanol (Sigma) at room temperature to prepare a clear stock solution. The solution was filtered through a 0.22 μm Millex-GP SLGPR33RB filter (Millipore) and then diluted with physiological saline (National Medicine Standard H11021190, 0.9 g/100 mL) to achieve a final concentration of 19.2 mg/mL (2.5% tribromoethanol). The working solution was incubated at 37°C for 4 hours, shaken, and stored at 4°C in the dark. For tumor induction, 160 μL (307 mg/kg) of 2.5% tribromoethanol was administered intraperitoneally to induce anesthesia. Once the mice were fully anesthetized, they were positioned in a sterile work area in a supine position, and the area was disinfected with povidone-iodine. A small incision (approximately 0.5 cm) was made above the right fourth mammary gland using ophthalmic scissors. The skin was gently lifted with a cotton swab to expose the mammary fat pad, and 40 μL of a serum-free suspension containing 5 × 10^5 4T1 cells was injected into the fat pad. Tumor volume was measured using calipers, and the volume was calculated using the formula: Volume (mm^3) = (Width^2 × Length)/2.

When the tumor volume reached 100 mm^3, treatment commenced. In the *in vivo* experiments using TOE, the low-dose (100 mg/kg) and high-dose (200 mg/kg) monotherapy groups were administered TOE daily by oral gavage. The combination therapy group received 5 mg/kg PD-1/PD-L1 inhibitor 2 via intraperitoneal injection every two days for a total of 14 days. In the experiments using commercial compounds, the monotherapy groups received daily intraperitoneal injections of ICGA-A (10 mg/kg) or every two days of CRA (20 mg/kg). The combination therapy group received 5 mg/kg PD-1/PD-L1 inhibitor 2 via intraperitoneal injection every two days for 14 days.

### 2.9 Flow cytometry

On day 15 of treatment, mice were euthanized using 2.5% isoflurane anesthetic. Tumors were carefully excised and transferred into enzyme digestion solution containing collagenase IV (2 mg/mL, Thermo Fisher Scientific), DNase I (0.1 mg/mL, Thermo Fisher Scientific), and RPMI-1640 medium (Procell) to degrade extracellular matrix tissue and remove impurities. Red blood cells were then eliminated using red blood cell lysis buffer (Biyuntian Biotechnology). Lymphocytes were isolated using Percoll (Solarbio) density gradient centrifugation. The resulting cell pellet was resuspended in PBS (Procell), and a single-cell suspension suitable for flow cytometry analysis was prepared.

The single-cell suspension was aliquoted into two tubes for different fluorescent antibody labeling. One tube was labeled with F4/80, CD11b, and CD45.2 antibodies, while the other tube was labeled with CD3e, CD4, CD8a, CD279 (PD-1), CD223 (LAG-3), and CD366 (TIM3) antibodies, all obtained from Thermo Fisher Scientific. Antibodies used here are listed in **Supplementary Table S1**. Flow cytometry (FACS) analysis was performed to assess the antitumor immune response, with 500,000 cells collected for evaluation. Flow cytometry data were analyzed using FlowJo software. Initial gating was performed based on forward scatter (FSC) and side scatter (SSC) plots. Immunophenotyping analysis was conducted using multiple fluorescence channels. The gating strategy is outlined in **Supplementary Figure S2**.

### 2.10 Network pharmacology

The relevant targets of nine compounds identified in TOE were obtained using SwissADME and SwissTargetPrediction. Interaction standardization between compounds and targets was achieved by processing target information from the Uniprot protein database. Using “triple-negative breast cancer” as the keyword, potential therapeutic targets were identified in the GeneCards, OMIM, and DisGeNET databases, with targets scoring above the median selected as potential “disease targets” for TNBC.

### 2.11 Transcriptome sequencing

Cells were seeded in 10 cm culture dishes and treated with DMSO, ICGA-A, and CRA for 24 hours in a 5% CO_2_ environment at 37°C. Following treatment, cells were collected using Trizol (Vazyme). RNA quality control, library construction, and sequencing were performed by GeneWiz Inc. GO and KEGG analyses were conducted using the DAVID v6.8 database, with results visualized through bar and bubble charts generated on the Bioinformatics platform.

### 2.12 Molecular docking

The compound structure files were obtained from the PubChem database. The three-dimensional structure of the FAK protein (PDB code: 6dj6) was retrieved from the Protein Data Bank (PDB). Molecular docking and virtual screening were performed using AutoDock Vina, and the visualizations were generated with PyMOL.

### 2.13 Chemicals

PD-1/PD-L1 inhibitor 2 (BMS202) were purchased from Selleck. Isochlorogenic acid A and Chicoric acid were purchased from TOPSCIENCE. All chemicals were dissolved in DMSO for the *in vivo* and *in vitro* studies.

### 2.14 Western blot assay

After the cells were treated with ICGA-A and CRA for 48 hours, they were lysed on ice using RIPA buffer (Solarbio) containing 1% PMSF and 1% phosphatase inhibitor (Proteintech). The protein concentration was measured using the Pierce BCA protein assay kit (Thermo Fisher Scientific). Equal amounts of protein (20 µg each) were loaded onto 10% SDS-PAGE (Vazyme) and FuturePAGE™ 4%–12% 12 Wells (Ace Biotechnology) and then transferred to a PVDF membrane (Millipore). The membrane was blocked at room temperature with 5% nonfat milk and then incubated overnight with the primary antibody at 4˚C. The membrane was washed with TBST, followed by incubation with the secondary antibody at room temperature. The protein signals in the BG-gdsAUTO730 were detected using a high-sensitivity ECL chemiluminescent detection kit (Proteintech). Original images of the Western blot analysis from three independent experiments are shown in **Supplementary Figure S3**. Antibodies used here are listed in **Supplementary Table S2**.

### 2.15 Statistical analysis

Data were collected from independent experiments and expressed as the mean ± SEM. All graphs and analyses were generated using GraphPad Prism software. The significance among multiple (three or more) groups was compared using one-way ANOVA analysis, and differences between the different groups were analyzed by a Student’s *t*-test.

## 3 Results

### 3.1 Enhancement of PD-1/PD-L1 inhibitor 2-mediated tumor growth suppression by TOE in the 4T1 mouse model of TNBC

**Figure 1:**
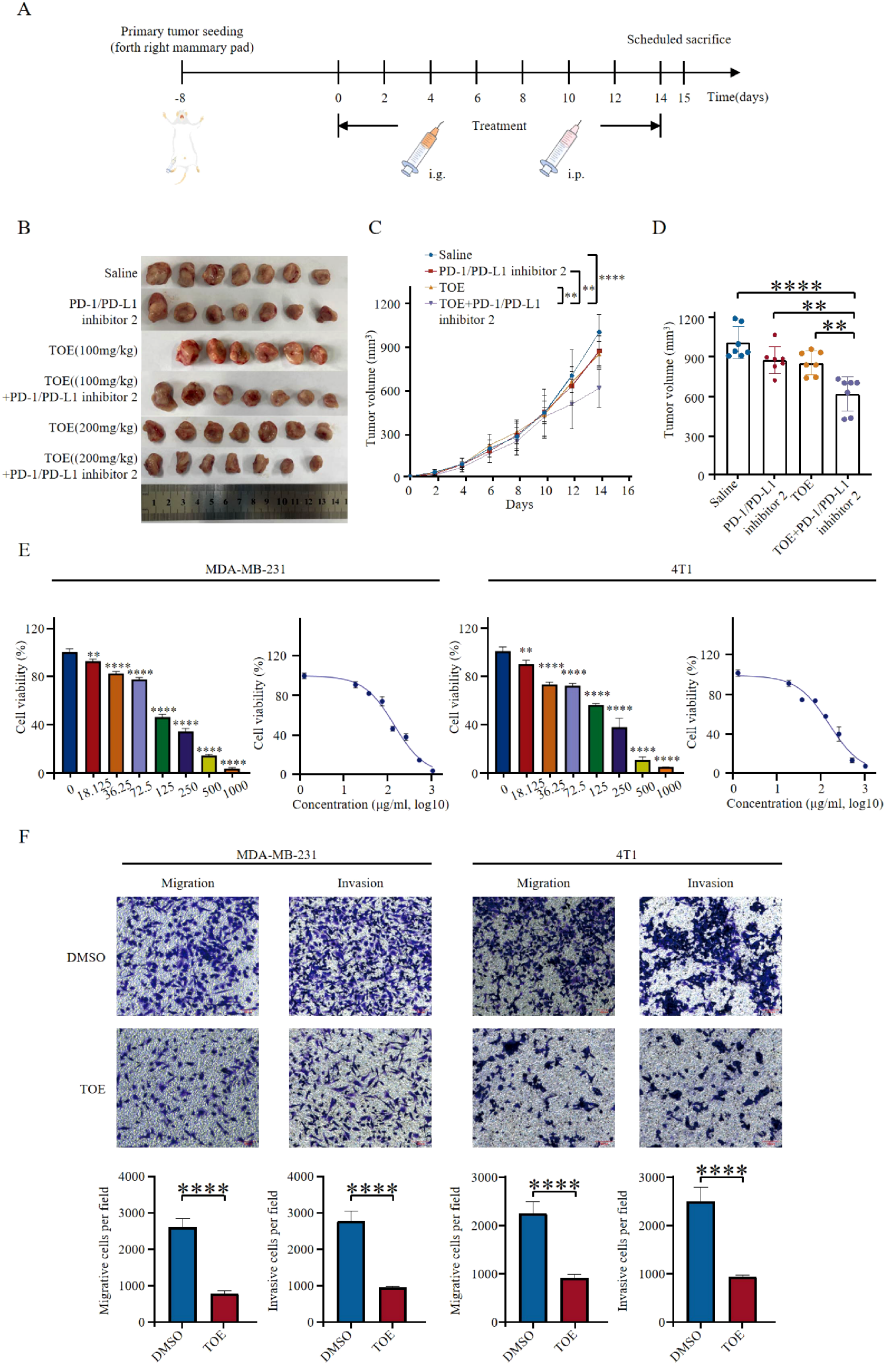
TOE combined with PD-1/PD-L1 inhibitor 2 suppresses tumor growth. **(A)** Illustration of animal models. 4T1 cells were injected into the mammary fat pads of mice. Animals were administered when the volumes of tumors were about 100 mm^3. **(B)** Diagrammatic representation of tumor volume measurement. The figure illustrates the method used for tumor volume calculation, where caliper-based measurements of tumor length and width were recorded and applied to the formula: Volume = 1/2 × length × width^2. Additionally, representative photographs of excised tumors at the end of the treatment period are presented. **(C)** Tumor growth curves of 4T1 tumor-bearing mice treated with TOE or TOE combined with PD-1/PD-L1 inhibitor 2 over a 14-day period. The average tumor volume was measured every two days and plotted over time to evaluate the antitumor efficacy of different treatment regimens. **(D)** Tumor sizes at day 14. The data are expressed as the mean ± SEM, with statistical significance calculated using one-way ANOVA. **p < 0.01, ***p < 0.001, ****p < 0.0001. **(E)** TOE inhibited cell proliferation on MDA-MB-231 and 4T1. Data are expressed as the mean ± SEM (n = 3). Statistical significances were calculated via Student’s *t*-test. **p < 0.01 and ***p < 0.001, ****p < 0.0001. **(F)** Transwell migration and invasion assay of MDA-MB-231 and 4T1 cells with TOE treatment for 24 h Representative images from randomly selected fields of transwell inserts are shown on the up side, and quantitative data are shown on the down side. Scalebar = 100 μm. Cell numbers were calculated and are expressed as the mean ± SEM of three independent experiments. **p < 0.01,***p < 0.001, ****p < 0.0001 vs. DMSO, as determined by unpaired *t*-tests, were regarded as significant.

To evaluate the effect of TOE on PD-1/PD-L1 inhibitor 2-mediated suppression of tumor growth in the 4T1 mouse model and its impact on TNBC immunotherapy, we established a 4T1 mouse model. Mice were randomly assigned to six different treatment groups: PD-1/PD-L1 inhibitor 2 alone, TOE (100 mg/kg), TOE (200 mg/kg), TOE (100 mg/kg) combined with PD-1/PD-L1 inhibitor 2, TOE (200 mg/kg) combined with PD-1/PD-L1 inhibitor 2, and saline control. Treatment was initiated on Day 0 when the tumor volume reached 100 mm^3. In the TOE (100 mg/kg) monotherapy group, 100 mg/kg of TOE was administered orally once daily from Day 0 to Day 14. In the TOE (200 mg/kg) monotherapy group, 200 mg/kg of TOE was administered orally once daily from Day 0 to Day 14. In the combination therapy groups, TOE was administered orally once daily from Day 0 to Day 14, while PD-1/PD-L1 inhibitor 2 (5 mg/kg) was administered intraperitoneally every 48 hours on Days 0, 2, 4, 6, 8, 10, 12, and 14 throughout the treatment period. For consistency, the PD-1/PD-L1 inhibitor 2 monotherapy group followed the same dosing schedule, receiving a single intraperitoneal injection of 5 mg/kg every 48 hours, ensuring a comparable treatment frequency across groups (**Figure 1A**). TOE (200 mg/kg) exhibited a more pronounced antitumor effect compared to the low-dose TOE (100 mg/kg), indicating a dose-dependent antitumor activity. Therefore, in subsequent analyses, TOE refers to the high-dose TOE (200 mg/kg) (**Figure 1B**). The combined use of PD-1/PD-L1 inhibitor 2 and TOE significantly inhibited tumor progression compared to TOE or PD-1/PD-L1 inhibitor 2 alone (**Figures 1C, D**). Compared with the control group, the combination of TOE and PD-1/PD-L1 inhibitor 2 did not cause significant changes in body weight or the visceral indices of the spleen, liver, or lungs, indicating that the combination therapy did not induce systemic toxicity (**Supplementary Figure S4**). Taken together, these findings suggest that TOE can enhance PD-1/PD-L1 inhibitor 2-mediated suppression of tumor growth in TNBC *in vivo*.

*In vitro* experiments were conducted using TNBC cell lines MDA-MB-231 and 4T1. Cells were treated with different concentrations of TOE, and cell viability was assessed using CCK-8 analysis. Results showed a dose-dependent decrease in cell viability, indicating that TOE has cytotoxic effects (**Figure 1E**). The 24-hour IC50 values for MDA-MB-231 and 4T1 cells were 135.9µg/mL and 150.2µg/mL, respectively. Furthermore, Transwell migration and Matrigel invasion assays indicated that TOE significantly reduced the migration and invasion capabilities of TNBC cells (**Figure 1F**).

### 3.2 Combination of PD-1/PD-L1 inhibitors 2 and TOE enhances tumor lymphocyte infiltration and improves the efficacy of ICB therapy

**Figure 2:**
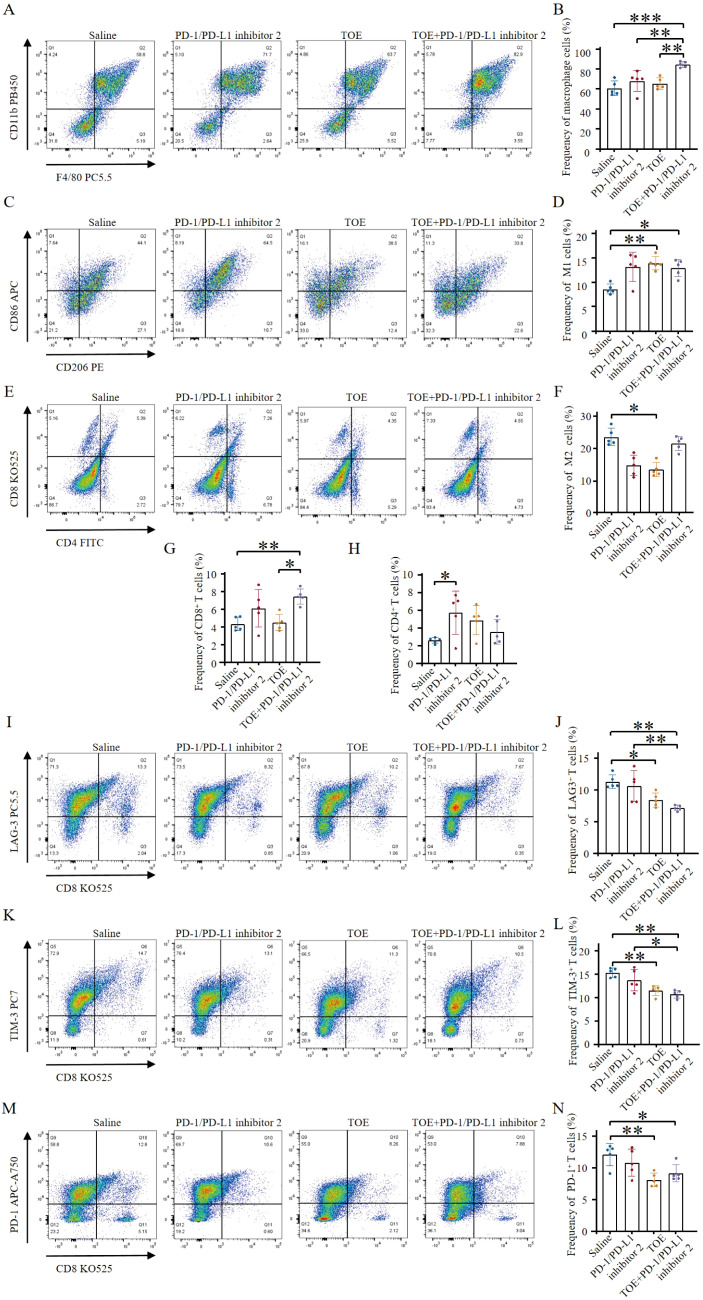
TOE combined with PD-1/PD-L1 inhibitor 2 immunotherapy promote immune stimulation of *in situ* 4T1 breast tumors. **(A)** Representative flow cytometry plots show tumor-associated macrophages (TAMs) (CD45.2+, CD11b+, F4/80+) following different treatments. **(B)** Tumor-associated macrophage levels were quantified by flow cytometry analysis. **(C)** Representative flow cytometry plots also show M1-like TAMs (CD11b+, F4/80+, CD86+) and M2-like TAMs (CD11b+, F4/80+, CD206+) after different treatments. **(D)** The levels of M1-type macrophages were quantified by flow cytometry analysis. **(E)** Representative flow cytometry plots showing tumor immune cells after different treatments, including CTLs (CD45+, CD3+, CD8+) and Th cells (CD45+, CD3+, CD4+). **(F)** The levels of M2-type macrophages were quantified by flow cytometry analysis. **(G, H)** The levels of CTLs and Th cells were quantified by flow cytometry analysis (n = 5). **(I, J)** Representative flow cytometry plots showing levels of LAG-3+ exhausted T cells (CD3+, CD8+, LAG-3+) following different treatments. Flow cytometry analysis quantified the levels of LAG-3+ exhausted T cells (n = 5). **(K, L)** Representative flow cytometry plots depicting levels of TIM-3+ exhausted T cells (CD3+, CD8+, TIM-3+) after various treatments. Flow cytometry analysis quantified the levels of TIM-3+ exhausted T cells (n = 5). **(M, N)** Representative flow cytometry plots displaying levels of PD-1+ exhausted T cells (CD3+, CD8+, PD-1+) following different treatment regimens. Flow cytometry analysis quantified the levels of PD-1+ exhausted T cells (n = 5). Data are expressed as the mean ± SEM. Statistical significances were calculated via one-way ANOVA (n = 5), *p < 0.05, **p < 0.01, ***p < 0.001.

To assess the ability of combination therapy to enhance tumor lymphocyte infiltration and modulate the immune-suppressive tumor microenvironment in 4T1 tumors, flow cytometry results revealed that PD-1/PD-L1 inhibitor 2 monotherapy had a negligible effect on macrophage infiltration. In contrast, combination therapy with PD-1/PD-L1 inhibitor 2 and TOE significantly increased macrophage recruitment into the tumor microenvironment (**Figures 2A, B**). Both TOE monotherapy and its combination with PD-1/PD-L1 inhibitor 2 significantly elevated the frequency of M1-type macrophages within tumor-infiltrating lymphocytes (**Figures 2C, D**). Moreover, TOE treatment notably reduced the frequency of M2-type macrophages (**Figure 2F**). These findings suggest that TOE promotes macrophage polarization toward the M1 phenotype in PD-1/PD-L1 inhibitor 2 treatment, although its underlying mechanism requires further elucidation. Evaluation of effector T cells revealed that, compared to the saline control group, PD-1/PD-L1 inhibitor 2 monotherapy and TOE monotherapy had a limited impact on CD8+ T cell infiltration. However, combination therapy with PD-1/PD-L1 inhibitor 2 and TOE significantly increased the proportion of CD8+ T cells in the tumor microenvironment, while the proportion of CD4+ T cells remained unchanged (**Figures 2E, G, H**).

Reversing T cell exhaustion is crucial for revitalizing the antitumor immune response, aiding the immune system in recognizing and eliminating tumor cells. Monotherapy with PD-1/PD-L1 inhibitor 2 did not significantly affect the antitumor activity of LAG-3+, TIM-3+, or PD-1+ T cell subsets. However, monotherapy with TOE and combination therapy with PD-1/PD-L1 inhibitor 2 and TOE significantly reduced the frequency of exhausted LAG-3+, TIM-3+, and PD-1+ T cells. Notably, the combination therapy more effectively reversed the exhaustion of LAG-3+ and TIM-3+ T cells, thereby enhancing the antitumor activity of T cells (**Figures 2I-N**). These results indicate that the combination of TOE and PD-1/PD-L1 inhibitor 2 not only enhances the antitumor effects of PD-1/PD-L1 inhibitor 2 therapy but also limits immune escape by tumors and improves the tumor microenvironment, thereby increasing the overall efficacy of cancer treatment. This combination therapy can reprogram the tumor microenvironment from a “cold” to a “hot” state, thereby activating immune cells to eliminate tumor cells and significantly improving the efficacy of immunotherapy.

### 3.3 Identification of constituents in TOE

**Figure 3:**
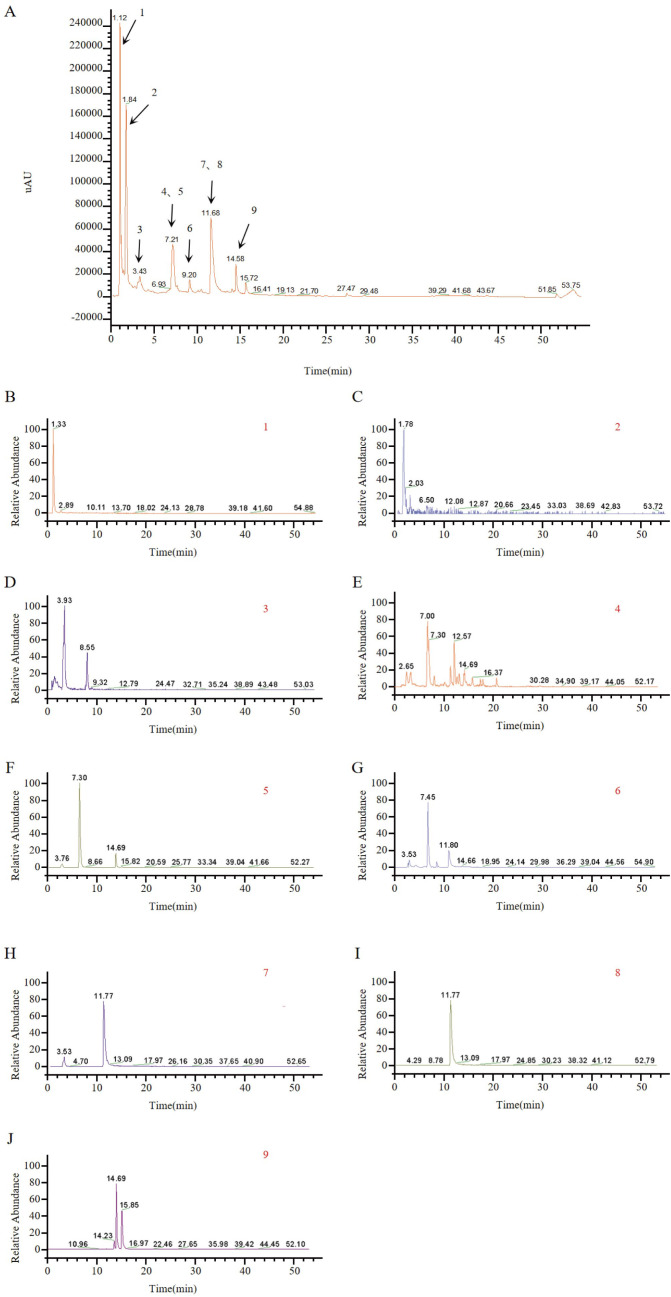
Identification of TOE components by UPLC-MS. **(A)** Total ion chromatograms, peaks are numbered according to **Table 1**. **(B)** MS spectrum of p-Coumaric acid. **(C)** MS spectrum of Gallic acid. **(D)** MS spectrum of Syringic acid. **(E)** MS spectrum of Ferulic acid. **(F)** MS spectrum of Chlorogenic acid. **(G)** MS spectrum of Caffeic acid. **(H)** MS spectrum of Caftaric acid. **(I)** MS spectrum of Chicoric acid. **(J)** MS spectrum of Isochlorogenic acid A.

Compounds of the *TOE* were identified using UPLC-Q-Orbitrap MS, and nine major components were obtained. The UPLC-Q-Orbitrap MS total ion chromatograms of the TOE are shown in **Figure 3A**. The mass spectra of these nine components were given in **Figures 3B-I**. The detailed information of the identified constituents in TOE was summarized in **Table 1**.

**Table 1:** MS data of constituents identified from TOE.

Peak NO.	name	Molecular foume	m/z	t_R_/min	Ion selected
1	p-Coumaric acid	C_9_H_8_O_3_	165.0552	1.33	[M+H]^+^
2	Gallic acid	C_7_H_6_O_5_	169.0137	1.78	[M-H]^-^
3	Syringic acid	C_9_H_10_O_5_	197.045	3.93	[M-H]^-^
4	Ferulic acid	C_10_H_10_O_4_	193.0501	7.00	[M-H]^-^
5	Chlorogenic acid	C_16_H_18_O_9_	353.0873	7.30	[M-H]^-^
6	Caffeic acid	C_9_H_8_O_4_	179.0344	7.45	[M-H]^-^
7	Caftaric acid	C_13_H_12_O_9_	311.0403	11.77	[M-H]^-^
8	Chicoric acid	C_22_H_18_O_12_	473.072	11.77	[M-H]^-^
9	Isochlorogenic acid A	C_25_H_24_O_12_	515.119	14.69/15.85	[M-H]^-^

### 3.4 Network pharmacology-based speculation on the potential regulatory mechanisms of TOE’s active components in TNBC

**Figure 4:**
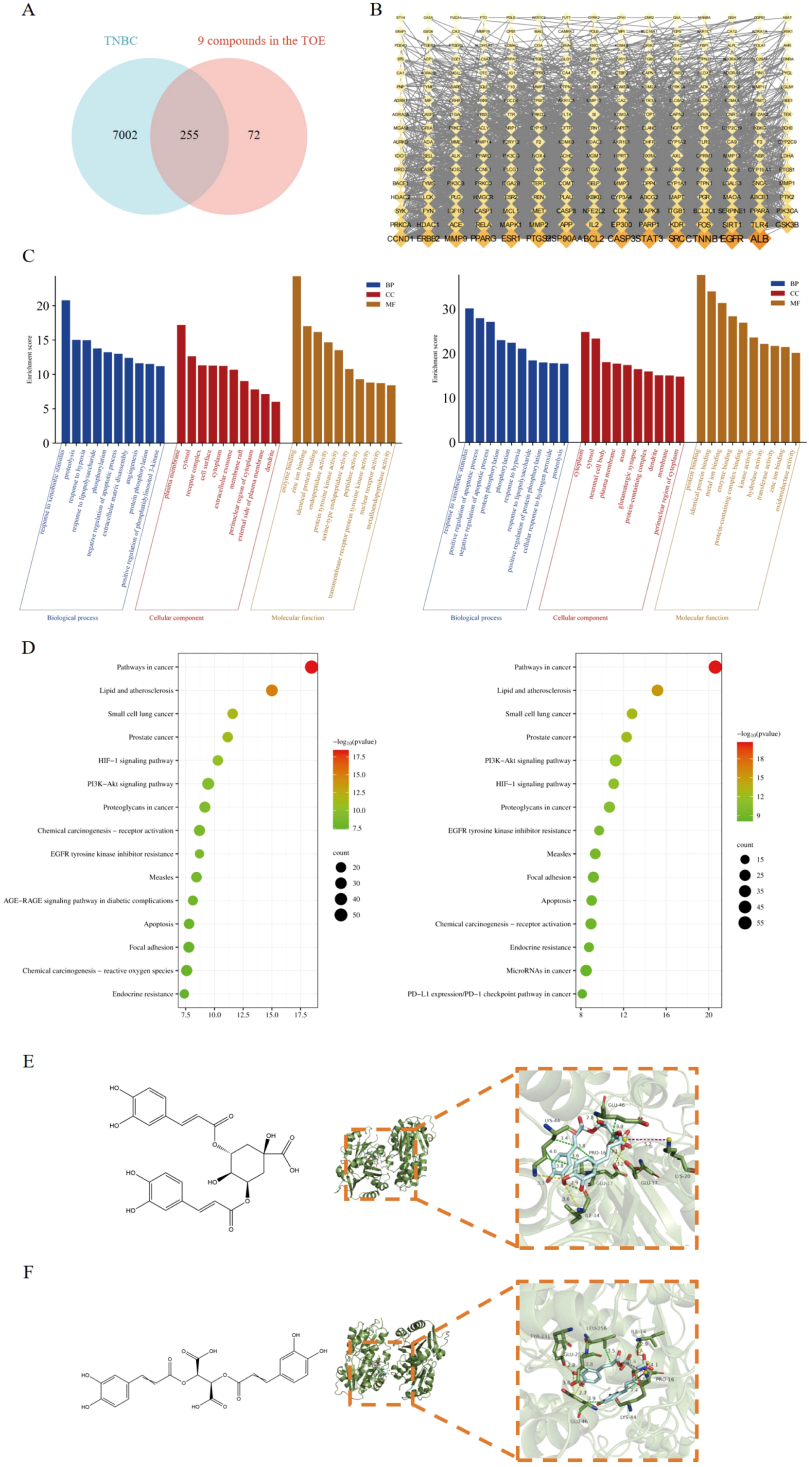
Network pharmacology predicted the core targets and the pathway analysis of the mechanism of the nine active compounds of TOE in TNBC. **(A)** Venny diagram of the overlapping targets of 9 compounds in the TOE and TNBC. **(B)** Compounds-disease-targets network. **(C)** The top 10 GO terms in the BP, CC, and MF classifications of overlapping genes with Homo sapiens (left) and Mus musculus (right). The x-axis represents the enriched terms, and the y-axis represents the enrichment score. **(D)** Top 20 KEGG pathways. KEGG pathway enrichment for the overlapping genes with Homo sapiens (left) and Mus musculus (right). The x-axis represents the gene ratio (*p <* 0.05), and the y-axis represents enriched terms. **(E)** The molecular structure of ICGA-A and the interaction between the FAK domain and the potential crystal structure of ICGA-A based on molecular docking simulations. **(F)** The molecular structure of CRA and the interaction between the FAK domain and the potential crystal structure of CRA based on molecular docking simulations. Yellow dashed lines represent hydrogen bonds; green dashed lines represent hydrophobic interactions; and purple dashed lines represent salt bridges.

SwissADME and SwissTargetPrediction were used to identify 327 potential targets for nine compounds meeting pharmacokinetic standards. The GeneCards database identified 7,257 TNBC-related gene targets. A cross-analysis between the 327 TOE-related complementary targets and the 7,257 TNBC-related targets revealed 255 overlapping genes (**Figure 4A**). Cytoscape 3.9.1 was employed to construct a network linking compounds, targets, and disease, displaying the names of 255 genes involved in drug-disease interactions (**Figure 4B**).

GO functional analysis and KEGG pathway enrichment were used to predict potential regulatory pathways through which TOE may exert anti-TNBC effects via these 255 targets. **Figure 4C** presents several enriched terms, including phosphorylation, extracellular matrix disassembly, cell surface, and protein tyrosine kinase activity, suggesting that TOE may exert anti-TNBC effects through these biological processes. Additionally, as shown in **Figure 4D**, several signaling pathways involved in tumorigenesis and tumor progression were enriched, including “receptor activation”, “cancer pathways”, “focal adhesion”, “apoptosis”, and the “PI3K/AKT signaling pathway”. These findings are closely related to signal transduction via cell surface receptors, suggesting that TOE may exert antitumor effects through these critical pathways and targets, including the PI3K/AKT signaling pathway. Further molecular docking studies and experimental validations will be conducted to confirm these predictions. FAK serves as an upstream regulatory factor of the PI3K/AKT signaling pathway, directly affecting the function of this pathway. To assess whether these compounds in TOE can directly interact with FAK, molecular docking simulations were performed (**Table 2**). Results indicate that the nine compounds exhibit varying binding affinities with FAK, with ICGA-A and CRA demonstrating the strongest affinities (**Figures 4E, F**).

**Table 2:** The binding scores between FAK and the compounds from TOE.

Ranking	Ligands	Binding score (kcal/mol)
1	Chicoric acid	-8.2
2	Isochlorogenic acid A	-7.8
3	Chlorogenic acid	-7.6
4	Caftaric acid	-7.1
5	Caffeic acid	-6.7
6	Ferulic acid	-6.6
7	p-Coumaric acid	-6.5
8	Gallic acid	-6
9	Syringic acid	-5.9

### 3.5 ICGA-A and CRA from TOE suppress TNBC tumor growth in mice and inhibit cellular behaviors associated with malignancy *in vitro*

**Figure 5:**
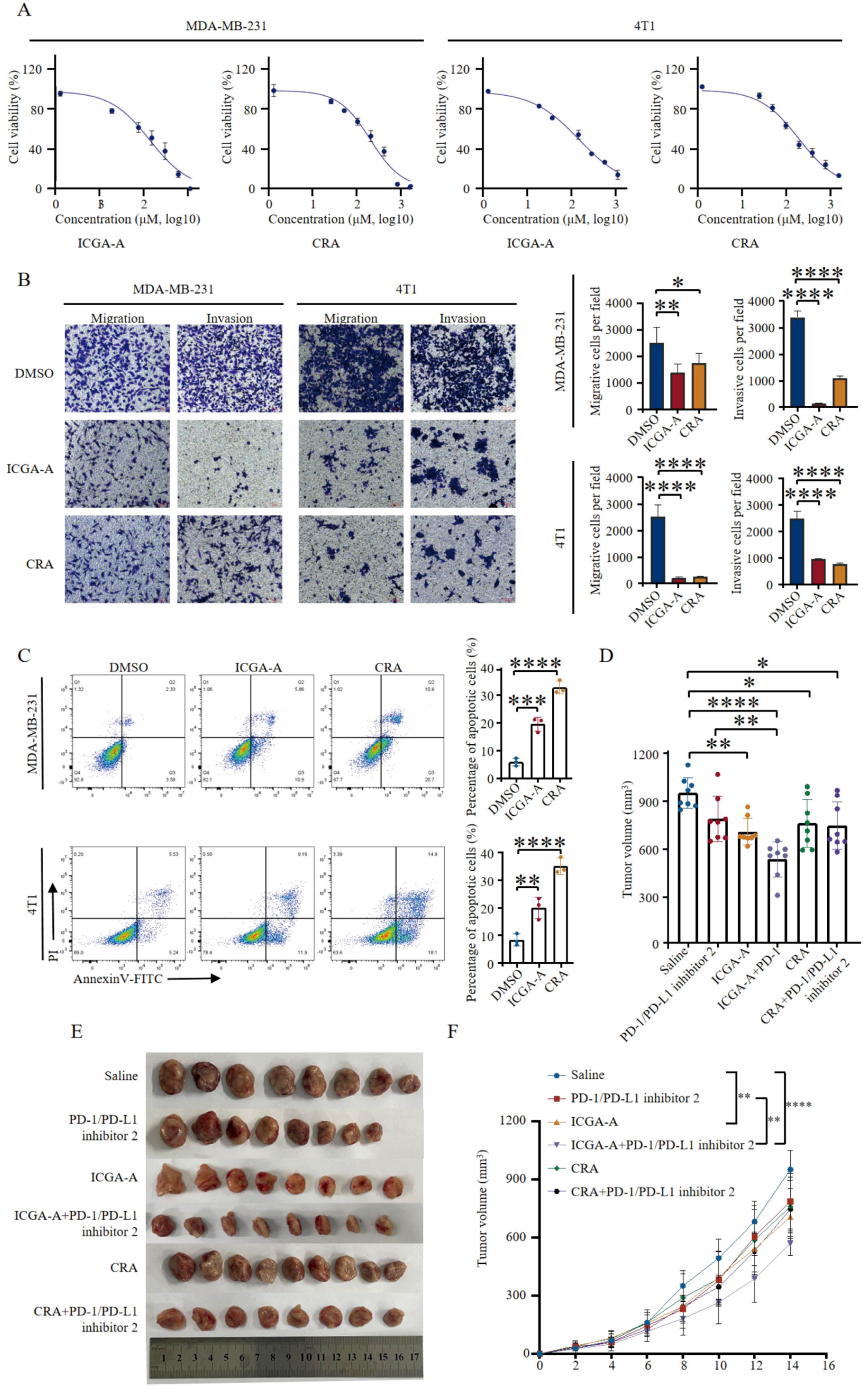
ICGA-A and CRA inhibited tumor growth in breast cancer mouse models. **(A)** Inhibition of growth by ICGA-A and CRA in MDA-MB-231 and 4T1 cells for 24 h Cell viability was monitored by CCK8 assay. The percentage of viability was calculated as the following formula: (viable cells)%=(OD of drug-treated sample/OD of untreated sample)×100. mean ± SEM (Student’s *t*-test). **(B)** Transwell migration and invasion assay of MDA-MB-231 and 4T1 cells with ICGA-A and CRA treatment for 24 h Representative images from randomly selected fields of transwell inserts are shown on the left side, and quantitative data are shown on the right side. Scalebar = 100 μm. Cell numbers were calculated and are expressed as the mean ± SEM of three independent experiments. *p < 0.05, **p < 0.01, ***p < 0.001, ****p < 0.0001, as determined by unpaired *t*-tests, were regarded as significant. **(C)** ICGA-A and CRA induced the apoptosis in MDA-MB-231 and 4T1 cells. The induction of apoptosis was detected by Annexin V-FITC/PI double staining assay. The apoptotic cell death was quantified as Annexin V+ (both PI-negative and PI-positive) cells. mean ± SEM (Student’s *t*-test) (n =3). **(D)** Diagrammatic representation of tumor volume measurement. **(E)** ICGA-A and CRA reduced the tumor growth in the 4T1 BALB/C mouse model; volume (mm^3^) = [width^2^ (mm^2^) × length (mm)]/2. Tumor volumes were measured every 2 days (n = 8). **(F)** Tumor sizes at day 14 (n = 8). Data are expressed as the mean ± SEM. Statistical significances were calculated via one-way analysis of variance (ANOVA).*p <05, **p < 0.01, ***p < 0.001, ****p < 0.0001.

The highest-scoring bioactive compounds in TOE, ICGA-A and CRA, exhibited binding energies of -8.2 kcal/mol and -7.8 kcal/mol, respectively. These two compounds were subsequently selected for *in vitro* validation. CCK-8 assays demonstrated that treatment with ICGA-A and CRA significantly reduced cell viability, inhibited cell proliferation, and slowed cell growth (**Figure 5A**). The 24-hour IC50 values of ICGA-A for MDA-MB-231 and 4T1 cells were 135.8µM and 154.9µM, and the 24-hour IC50 values of CRA for MDA-MB-231 and 4T1 cells were 193.8µM and 198.6µM, respectively. Transwell assays indicated that ICGA-A and CRA significantly decreased the migration and invasion capabilities of MDA-MB-231 and 4T1 cells (**Figure 5B**). Flow cytometry was utilized to assess whether ICGA-A and CRA induced apoptosis. The results revealed that treatment with ICGA-A and CRA led to a substantial increase in the frequency of Annexin V-positive cells, effectively promoting apoptosis (**Figure 5C**).

To evaluate the potential of ICGA-A and CRA to inhibit TNBC tumor proliferation *in vivo*, a 4T1 mouse tumor model was established. Mice were randomly assigned to six groups, each receiving a different treatment regimen: PD-1/PD-L1 inhibitor 2 monotherapy, ICGA-A (10 mg/kg), CRA (20 mg/kg), combined ICGA-A with PD-1/PD-L1 inhibitor 2, combined CRA with PD-1/PD-L1 inhibitor 2, and a saline control group. Compared to the control group, both ICGA-A and CRA significantly inhibited the growth of 4T1 TNBC tumors. Furthermore, the combination of ICGA-A and PD-1/PD-L1 inhibitor 2 exhibited superior tumor-suppressive effects compared to PD-1/PD-L1 inhibitor 2 alone, suggesting the potential of this combination as a therapeutic strategy for TNBC (**Figures 5D-F**). In the combination treatment group, body weight and the organ indices of the spleen, liver, and lung remained unchanged, indicating the safety of this therapeutic approach (**Supplementary Figure S5**).

### 3.6 Combination therapy of ICGA-A and CRA with PD-1/PD-L1 inhibitor 2 enhances antitumor immune response

**Figure 6:**
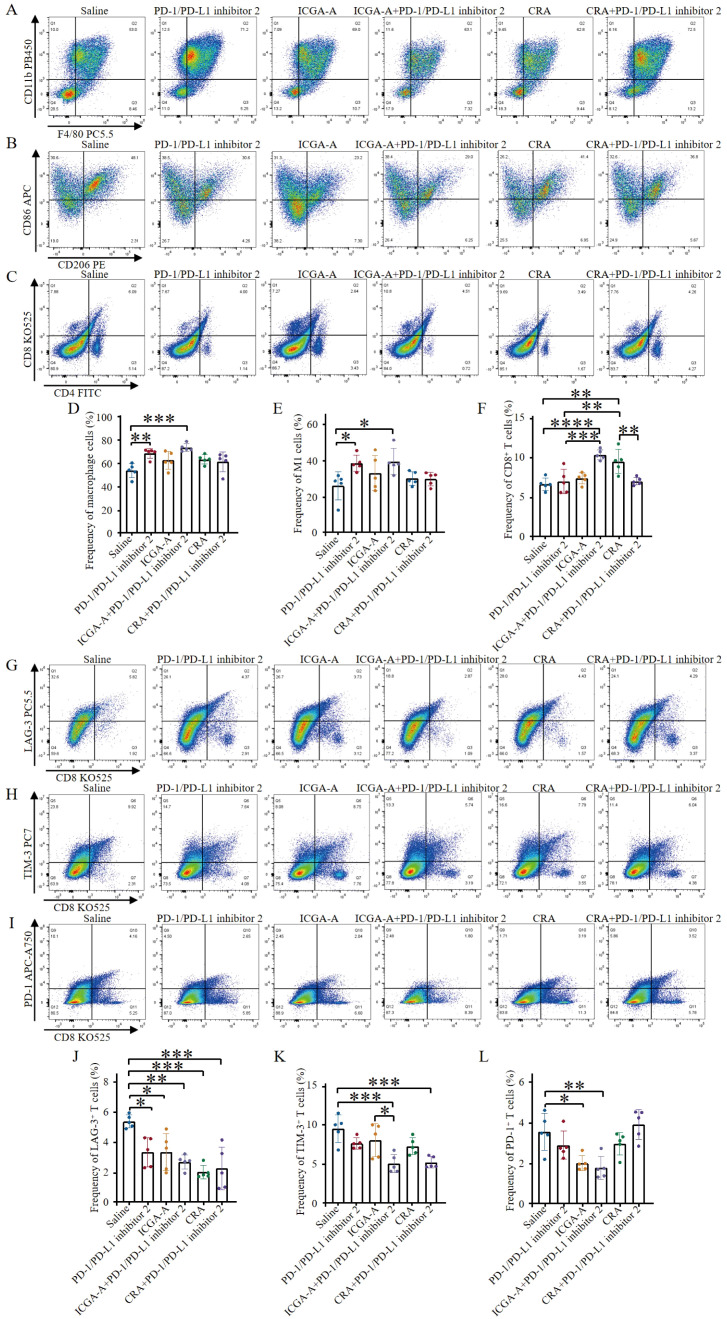
ICGA-A combined with PD-1/PD-L1 inhibitor 2 improves the immune microenvironment and promotes lymphocyte infiltration. **(A)** Representative flow cytometry plots showing tumor-associated macrophages (TAMs) (CD45.2+, CD11b+, F4/80+) were obtained after different treatments. **(B)** Representative flow cytometry plots displaying tumor-infiltrating immune cells after different treatments, including M1-like TAMs (CD45.2+, CD11b+, F4/80+, CD86+) and M2-like TAMs (CD45.2+, CD11b+, F4/80+, CD206+). **(C)** Representative flow cytometry plots showing tumor immune cells after different treatments, including CTLs (CD45+, CD3+, CD8+) and Th cells (CD45+, CD3+, CD4+). **(D)** The levels of TAMs were quantified through flow cytometry analysis (n = 5). **(E)** The proportions of tumor-associated M1-type macrophages were quantified through flow cytometry analysis (n = 5). **(F)** The levels of CTLs were quantified by flow cytometry analysis (n = 5). **(G)** Representative flow cytometry plots demonstrating tumor-infiltrating LAG-3+ exhausted T cells (CD3+, CD8+, LAG-3+) after different treatments. **(H)** Representative flow cytometry plots demonstrating tumor-infiltrating TIM-3+ exhausted T cells (CD3+, CD8+, TIM-3+) after different treatments. **(I)** Representative flow cytometry plots demonstrating tumor-infiltrating PD-1+ exhausted T cells (CD3+, CD8+, PD-1+) after different treatments. **(J)** Flow cytometry analysis quantified the levels of LAG-3+ exhausted T cells (n = 5). **(K)** Flow cytometry analysis quantified the levels of TIM-3+ exhausted T cells (n = 5). **(L)** Flow cytometry analysis quantified the levels of PD-1+ exhausted T cells (n = 5). Data are expressed as the mean ± SEM. Statistical significances were calculated via one-way ANOVA, *p < 0.05, **p < 0.01, ***p < 0.001 and ****p < 0.0001 vs. DMSO.

The reprogramming effects of active compound monomers on the polarization of tumor-associated macrophages (TAMs) and their potential to modulate the immunosuppressive microenvironment were assessed by quantifying the frequencies of total macrophages, M1-type TAMs, and M2-type TAMs using flow cytometry. The results indicated that combination treatment with ICGA-A and PD-1/PD-L1 inhibitor 2 significantly increased the frequency of anti-tumor M1-type TAMs, while having no significant effect on immunosuppressive M2-type TAMs (**Figures 6B, E**). Moreover, compared to the control group, the frequency of total tumor-associated macrophages was also significantly increased (**Figures 6A, D**). Analysis of effector T cells revealed that, compared to other groups, combination therapy with ICGA-A and PD-1/PD-L1 inhibitor 2 significantly increased the frequency of effector CD8+ T cells, surpassing the PD-1/PD-L1 inhibitor 2 monotherapy group. Additionally, the frequency of effector T cells in the CRA monotherapy group was also higher than that in the PD-1/PD-L1 inhibitor 2 monotherapy group (**Figures 6C, F**). Notably, none of the drug treatments had a significant impact on Treg levels (**Supplementary Figure S6**). These findings suggest that the ICGA-A combination therapy strategy can effectively enhance the immune response to immune checkpoint blockade (ICB) therapy.

This study also explored the effects of ICGA-A and CRA as monotherapy or in combination with PD-1/PD-L1 inhibitor 2 on T cell exhaustion. The results indicated that both compounds, whether used alone or in combination with PD-1/PD-L1 inhibitor 2, effectively reduced the frequency of exhausted T cells expressing LAG-3 (**Figures 6G, J**). Combination treatment with both compounds exhibited a similar trend in reducing TIM-3 expression levels (**Figures 6H, K**). Both the ICGA-A monotherapy and its combination with PD-1/PD-L1 inhibitor 2 effectively reduced the frequency of PD-1-positive exhausted T cells (**Figures 6I, L**). These results not only highlight the potential immunomodulatory roles of ICGA-A and CRA in regulating T cell exhaustion but also suggest that their combination with PD-1/PD-L1 inhibitor 2 may enhance T cell-mediated tumor immune responses. These findings provide robust experimental evidence for the development of novel tumor immunotherapy strategies, particularly those that utilize natural products to modulate the tumor microenvironment and enhance existing immunotherapies.

### 3.7 ICGA-A and CRA deregulate immunosuppression by inhibiting the FAK/PI3K/AKT/mTOR signaling pathway

**Figure 7:**
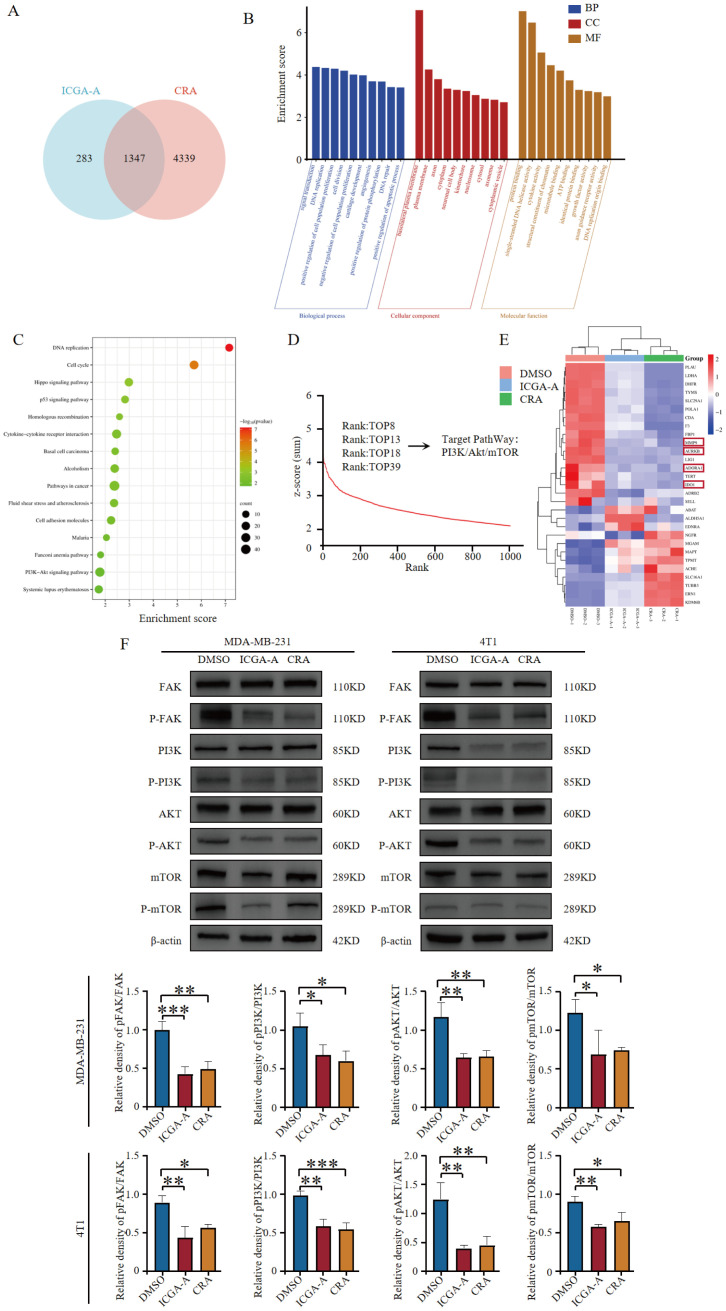
ICGA-A and CRA inhibited mRNA and protein expression levels of metabolic-associated proteins and pathways. **(A)** Venn diagram of DEGs in MDA-MB-231 cells treated with ICGA-A and CRA. **(B)** The top 10 GO terms in the BP, CC, and MF classifications of overlapping genes with MDA-MB-231. The x-axis represents the enriched terms, and the y-axis represents the enrichment score. **(C)** Top 20 KEGG pathways. KEGG pathway enrichment for the overlapping genes with MDA-MB-231. The x-axis represents the gene ratio (*p <* 0.05), and the y-axis represents the enriched terms. **(D)** The ranking of ICGA-A scored by HTS^2^ in a library of over 20,000 compounds. **(E)** Heatmap of the intersecting genes between RNA-seq and network pharmacology targets. **(F)** ICGA-A and CRA decreased the phosphorylation levels of FAK, PI3K, AKT, and mTOR. *p < 0.05, **p < 0.01, ***p < 0.001 vs. DMSO.

To further elucidate the molecular mechanisms by which ICGA-A and CRA synergize with PD-1/PD-L1 inhibitor 2 to exert antitumor immune functions, RNA sequencing (RNA-seq) was conducted. Cross-analysis of MDA-MB-231 cells treated with ICGA-A and CRA revealed 1,347 overlapping DEGs (*p <* 0.05) (**Figure 7A**). Cells treated with ICGA-A and CRA showed significant enrichment in the “Cancer pathway”, “VEGF pathway”, “PI3K-Akt signaling pathway”, “Focal adhesion pathway”, and “ECM-receptor interaction pathway” (**Figures 7B, C**). In compound library screening conducted using HTS^2^, ICGA-A showed strong specificity for the PI3K signaling pathway, ranking in the top 8 out of 20,000 compounds and targeting the PI3K/Akt/mTOR signaling pathway (**Figure 7D**).

By overlapping the 255 targets identified through network pharmacology analysis with the differentially expressed genes (DEGs) (*p <* 0.05) from ICGA-A and CRA-treated MDA-MB-231 cells, RNA-seq analysis demonstrated a similar trend for these compounds in suppressing antitumor immunity-related genes (e.g., IDO1, MMP9, ADORA1, and AURKB) (**Figure 7E**). ICGA-A and CRA significantly inhibited the expression of various antitumor immunity-related genes, thereby modulating the immune microenvironment. Western blot results indicated that ICGA-A and CRA reduced the phosphorylation levels of FAK, PI3K, AKT, and mTOR, suggesting that these compounds may be key bioactive components within TOE (**Figure 7F**).

## 4 Discussion

In this study, we demonstrate that TOE, when combined with a PD-1/PD-L1 inhibitor 2, effectively suppresses TNBC growth in a syngeneic mouse model. This combination not only increases the infiltration of M1 macrophages and CD8+ T cells but also reduces the population of exhausted T cells within the tumor microenvironment, suggesting its potential as a novel immunotherapeutic approach. Through bioactive compound analysis, we identified nine compounds in the TOE, with ICGA-A and CRA displaying the most potent anti-TNBC activity. Both compounds significantly inhibited TNBC growth *in vivo* when used in combination with a PD-1/PD-L1 inhibitor 2. Notably, ICGA-A demonstrated superior efficacy by markedly increasing M1 macrophage and CD8+ T cell levels while reducing exhausted T cells. CRA, although less effective in modulating CD8+ T cell infiltration, showed a similar trend in anti-tumor activity, primarily by enhancing M1 macrophage levels. Mechanistic studies revealed that these effects are mediated through the inhibition of the FAK/PI3K pathway.

Our findings highlight the potential of combining *Taraxacum officinale* derivatives, particularly ICGA-A and CRA, with PD-1/PD-L1 inhibitor 2 as a novel strategy to enhance the efficacy of immune checkpoint blockade in TNBC. This study provides a new therapeutic avenue by leveraging the synergistic effects of herbal compounds with modern immunotherapy, contributing to the ongoing search for effective combination treatments in cancer therapy.

This study provides robust evidence that ICGA-A, a bioactive compound derived from *Taraxacum officinale*, significantly enhance the efficacy of PD-1/PD-L1 inhibitor 2 by modulating the tumor microenvironment (TME) in TNBC. By promoting TAM polarization, enhancing CD8+ T cell infiltration, and inhibiting the FAK/PI3K/AKT/mTOR signaling axis, these compounds offer a compelling strategy for improving immune checkpoint blockade (ICB) outcomes in TNBC. Our results highlight the potential of integrating natural compounds with immune checkpoint inhibitors (ICIs), which may expand the therapeutic landscape for this highly aggressive malignancy.

The limited response rate to ICIs in TNBC has yet to be fully elucidated. A key contributing factor is the paucity of activated immune cells within the TME, characterizing many TNBC tumors as “cold” and thus less responsive to immunotherapy (16, 17). The immunosuppressive characteristics of the TME, particularly the presence of M2 macrophages and regulatory T cells (Tregs), further attenuate the antitumor response of the immune system. M2 macrophages facilitate tumor immune evasion by delivering immunosuppressive signals within the TME, whereas Tregs suppress the function of effector T cells by secreting immunosuppressive factors (18–21). Cytokines play a pivotal role in shaping the TME by influencing immune cell behavior and contributing to immune suppression. For example, interleukin-10 (IL-10) and transforming growth factor-beta (TGF-β) are secreted by M2 macrophages and Tregs, promoting an immunosuppressive environment that inhibits the activation of effector T cells (22–25). Additionally, pro-inflammatory cytokines such as TNF-α and IL-6 can exacerbate tumor progression by promoting chronic inflammation, further hindering the efficacy of immunotherapies (26, 27). Therefore, targeting cytokine-mediated pathways in the TME could potentially enhance the responsiveness of TNBC to ICIs by reversing immune suppression and activating antitumor immune responses.

Previous work has demonstrated that targeting the JAK1/STAT3 pathway with aurora kinase inhibitors increases the expression of Th1 chemokines such as CXCL10 and CXCL11, converting “cold” tumors into “hot” ones (4). This approach has been shown to improve ICI efficacy, and our current findings similarly indicate that ICGA-A can remodel the TME by promoting immune cell recruitment and antitumor immunity. Notably, the combination of ICGA-A and PD-1/PD-L1 inhibitor 2 significantly enhanced M1 macrophage polarization and CD8+ T cell infiltration while reducing T cell exhaustion. Through BLISS analysis (28, 29), it was found that the combination of ICGA-A and PD-1/PD-L1 inhibitor 2 resulted in a synergistic effect, while the combination of CRA and PD-1/PD-L1 inhibitor 2 exhibited an antagonistic effect. Although CRA alone reduced tumor volume, its combination with the PD-1/PD-L1 inhibitor did not show a significant difference compared to CRA alone. This lack of synergy may be attributed to differential interactions between CRA and immune cells in the tumor microenvironment. CRA is suggested to exert its anti-tumor effects by enhancing the infiltration of CD8+ T cells, but no significant enhancement was observed in the combination treatment. This phenomenon may be related to changes in PD-1+ exhausted T cells. Specifically, the adjusted *p*-values for the CRA and CRA+PD-1/PD-L1 inhibitor 2 groups, compared to the saline control group, were 0.3721 and 0.2800, respectively. Although these differences were not statistically significant, the observed trend suggests that CRA alone reduced the frequency of PD-1+ exhausted T cells, while the combination treatment increased this frequency. These findings indicate that the combination of CRA and PD-1/PD-L1 inhibitor 2 may alter T cell status, potentially exacerbating the exhaustion of PD-1+ T cells, instead of enhancing the immune regulatory effects of PD-1 blockade. This synergistic interaction between ICGA-A and PD-1/PD-L1 inhibitor 2 blockade suggests that ICGA-A may help overcome immune suppression within the TME. While CRA exhibited a similar trend, its effects, particularly on CD8+ T cell infiltration, were less pronounced. Nonetheless, both compounds demonstrated potent antitumor activity *in vivo*, suggesting their utility as adjuncts to ICI therapy.

Mechanistically, both ICGA-A and CRA inhibited the FAK/PI3K/AKT signaling pathway, as evidenced by reduced phosphorylation of key downstream proteins. FAK plays a critical role in tumor cell survival and immune evasion and has been implicated in cancer progression and metastasis across various malignancies (30–35). Our molecular docking analyses further supported the strong binding affinities of ICGA-A and CRA to FAK, suggesting that FAK inhibition is a key mechanism driving their antitumor effects. Beyond reducing tumor cell proliferation and migration, FAK inhibition also enhances immune cell infiltration, thereby augmenting the antitumor effects of ICIs (36–38).

Despite the promising findings, several challenges remain in the clinical translation of these compounds. While the anti-inflammatory properties of herbal medicines such as dandelion have been recognized in clinical practice, the complexity of their bioactive compounds and the interactions between these components present significant obstacles to standardization and a comprehensive understanding of their pharmacological effects. Although our *in vivo* results are encouraging, further investigations are required to elucidate the precise molecular targets and mechanisms of action of these compounds. Integrative omics plays a crucial role in clinical translation by analyzing gene expression, variations, and regulatory networks while incorporating environmental factors and systems biology approaches (39). This strategy facilitates the identification of key disease biomarkers and interaction networks, thereby advancing precision medicine and optimizing personalized therapeutic strategies. Future research should focus on optimizing the dosage and combination of these compounds to maximize therapeutic potential while minimizing toxicity. Furthermore, a deeper understanding of the interplay between local and systemic immune responses is essential to fully harness the therapeutic potential of ICGA-A in combination with PD-1/PD-L1 inhibitor 2 for TNBC treatment.

## Data Availability

The datasets presented in this study can be found in online repositories. The names of the repository/repositories and accession number(s) can be found below: https://www.ncbi.nlm.nih.gov/, PRJNA1215589.
