# Correction: Therapeutic potential of isochlorogenic acid A from *Taraxacum officinale* in improving immune response and enhancing the efficacy of PD-1/PD-L1 blockade in triple-negative breast cancer

**DOI:** 10.3389/fimmu.2025.1672972

**Published:** 2025-09-02

**Authors:** Tangyi Wang, Jingwei Sun, Li Wang, Yuxin Lin, Zhijing Wu, Qiangqiang Jia, Shoude Zhang, Juan An, Xueman Ma, Qiong Wu, Zhanhai Su, Haiyan Wang

**Affiliations:** ^1^ Department of Basic Medical Sciences, Qinghai University Medical College, Xining, Qinghai, China; ^2^ Department of Medical Laboratory, Qinghai Provincial People’s Hospital, Xining, Qinghai, China; ^3^ State Key Laboratory of Plateau Ecology and Agriculture, Qinghai University, Xining, Qinghai, China; ^4^ Research Center for High Altitude Medicine, Qinghai University, Xining, Qinghai, China; ^5^ Key Laboratory of the Ministry of High Altitude Medicine, Qinghai University, Xining, Qinghai, China

**Keywords:** immune checkpoint blockade, *Taraxacum officinale*, PD-1/PD-L1 inhibitor 2, tumor microenvironments, triple-negative breast cancer

There was a mistake in **Figure 7F** as published. The error was caused by the use of placeholder images during the typesetting process to maintain layout consistency; due to oversight, some placeholders were not replaced with the correct images. The corrected **Figure 7F** appears below.

**Figure 7:**
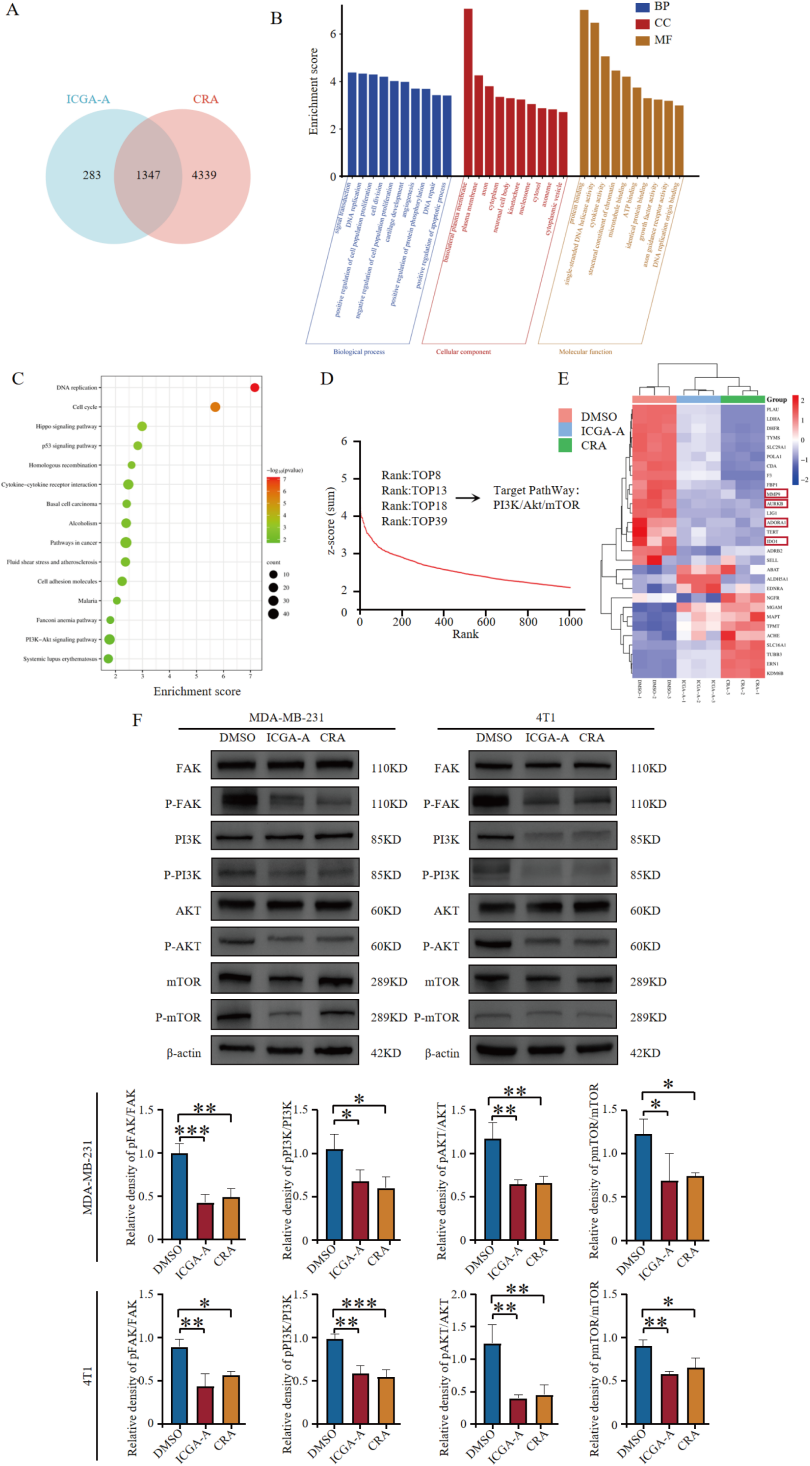
ICGA-A and CRA inhibited mRNA and protein expression levels of metabolic-associated proteins and pathways. **(A)** Venn diagram of DEGs in MDA-MB-231 cells treated with ICGA-A and CRA. **(B)** The top 10 GO terms in the BP, CC, and MF classifications of overlapping genes with MDA-MB-231. The x-axis represents the enriched terms, and the y-axis represents the enrichment score. **(C)** Top 20 KEGG pathways. KEGG pathway enrichment for the overlapping genes with MDA-MB-231. The x-axis represents the gene ratio (*p* < 0.05), and the y-axis represents the enriched terms. **(D)** The ranking of ICGA-A scored by HTS^2^ in a library of over 20,000 compounds. **(E)** Heatmap of the intersecting genes between RNA-seq and network pharmacology targets. **(F)** ICGA-A and CRA decreased the phosphorylation levels of FAK, PI3K, AKT, and mTOR. *p < 0.05, **p < 0.01, ***p < 0.001 vs. DMSO.

The original version of this article has been updated.

