# Supplementary material for: Therapeutic potential of isochlorogenic acid A from Taraxacum officinale in improving immune response and enhancing the efficacy of PD-1/PD-L1 blockade in triple-negative breast cancer
Source: Front Immunol. 2025 Mar 5;16:1529710. doi: 10.3389/fimmu.2025.1529710 (PMC11920172; doi:10.3389/fimmu.2025.1529710)
Supplement: Supplementary file 1 [file DataSheet1.docx]

Supplementary Material

# Supplementary Tables

**Supplementary Table1: Antibodies used in flow cytometry**

| Antibodies | Catalog No. | dilution ratio |
| --- | --- | --- |
| F4/80 Monoclonal Antibody (BM8),PerCP-Cyanine5.5 | 45-4801-82 | 1: 5 |
| CD11b Monoclonal Antibody (M1/70), eFluor™450 | 48-0112-82 | 1: 1 |
| CD45.2 Monoclonal Antibody (104), FITC | 11-0454-82 | 1: 4 |
| CD86 (B7-2) Monoclonal Antibody (GL1), APC | 17-0862-81 | 1: 1 |
| CD206 (MMR) Monoclonal Antibody (MR6F3), PE | 12-2061-80 | 1: 2 |
| CD3e Monoclonal Antibody (145-2C11), eFluor™ 450 | 48-0031-80 | 1: 5 |
| CD4 Monoclonal Antibody (GK1.5), FITC | 11-0041-82 | 1: 2 |
| CD8a Monoclonal Antibody (53-6.7),Brilliant Violet™ 480 | 414-0081-80 | 1: 5 |
| CD279 (PD-1) Monoclonal Antibody (J43), APC-eFluorTM780 | 47-9985-82 | 1: 5 |
| CD223 (LAG-3) Monoclonal Antibody，PerCP-eFluor™ 710 | 46-2231-82 | 1: 5 |
| CD366 (TIM3) Monoclonal Antibody (RMT3-23), PE-Cyanine7 | 25-5870-82 | 1: 5 |
| FOXP3 Monoclonal Antibody (FJK-16s), PE | 12-5773-80 | 1: 5 |
| CD25 Monoclonal Antibody (PC61.5), Alexa Fluor™ 700 | 56-0251-82 | 1: 5 |

**Supplementary Table2: Antibodies used in western blot**

| Antibodies | Catalog No. | Dilution ratio | Manufactor |
| --- | --- | --- | --- |
| Beta Actin Polyclonal antibody | 20536-1-AP | 1: 2000 | ProteinTech Group, Inc. |
| FAK Polyclonal antibody | 12636-1-AP | 1: 2000 | ProteinTech Group, Inc. |
| PI3 Kinase p85 Alpha Monoclonal antibody | 60225-1-Ig | 1:25000 | ProteinTech Group, Inc. |
| AKT Polyclonal antibody | 10176-2-AP | 1:6000 | ProteinTech Group, Inc. |
| mTOR Polyclonal antibody | 28273-1-AP | 1:5000 | ProteinTech Group, Inc. |
| Phospho-mTOR (Ser2448) Recombinant antibody | 80596-1-RR | 1:20000 | ProteinTech Group, Inc. |
| Multi-rAb HRP-Goat Anti-Mouse Recombinant Secondary Antibody (H+L) | RGAM001 | 1:5000 | ProteinTech Group, Inc. |
| Multi-rAb HRP-Goat Anti-Rabbit Recombinant Secondary Antibody (H+L) | RGAR001 | 1:5000 | ProteinTech Group, Inc. |
| Phospho-FAK (Tyr925) Antibody | 3284T | 1:1400 | Cell Signaling Technology, Inc. |
| Phospho-PI3 Kinase p85 (Tyr458)/p55 (Tyr199) Antibody | 4228T | 1:1000 | Cell Signaling Technology, Inc. |
| Phospho-Akt (Ser473) (D9E) XP® Rabbit mAb | 4060T | 1:2000 | Cell Signaling Technology, Inc. |

# Supplementary Figures

2.1 Supplementary Figue 1

**Supplementary Figue 1: TOE inhibits cell viability in TNBC cell lines.** (A) Effect of different concentrations and time points of TOE on cell viability. MDA-MB-231 and 4T1 cells were treated with various concentrations of TOE for 24, 48, and 72 hours. Cell viability was assessed using the CCK-8 assay. The data are presented as mean ± SEM. Statistical significance was determined using Dunnett’s multiple comparisons test, with P values indicated as follows: *p* > 0.05 = 0, *p* < 0.05 = 1, *p* < 0.01 = 2, *p* < 0.001 = 3, *p* < 0.0001 = 4. Statistical significance levels are indicated by the corresponding numbers.

2.2 Supplementary Figue 2


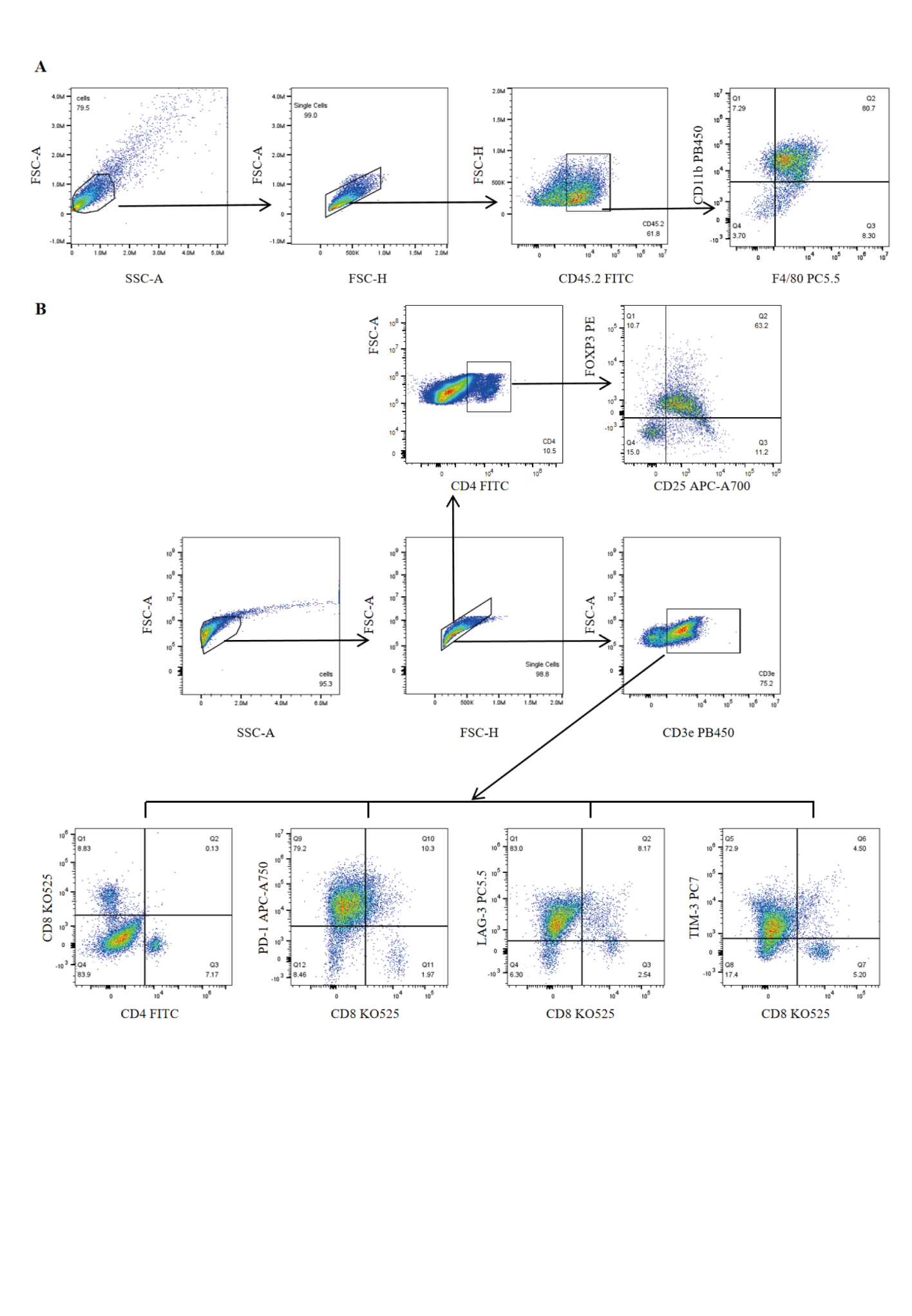


**Supplementary Figue 2: The gating strategy for flow cytometry of tumor-associated macrophages and tumor-infiltrating T cells.**

1. Flow cytometry gating strategies for M1-type and M2-type tumor-associated macrophages.
2. Flow cytometry gating strategy for analyzing the expression of tumor-infiltrating immune cell subsets, including Treg cells, CD4⁺ T cells, CD8⁺ T cells, and exhausted T cell populations characterized by LAG-3⁺, TIM-3⁺, and PD-1⁺ expression.

2.3 Supplementary Figue 3


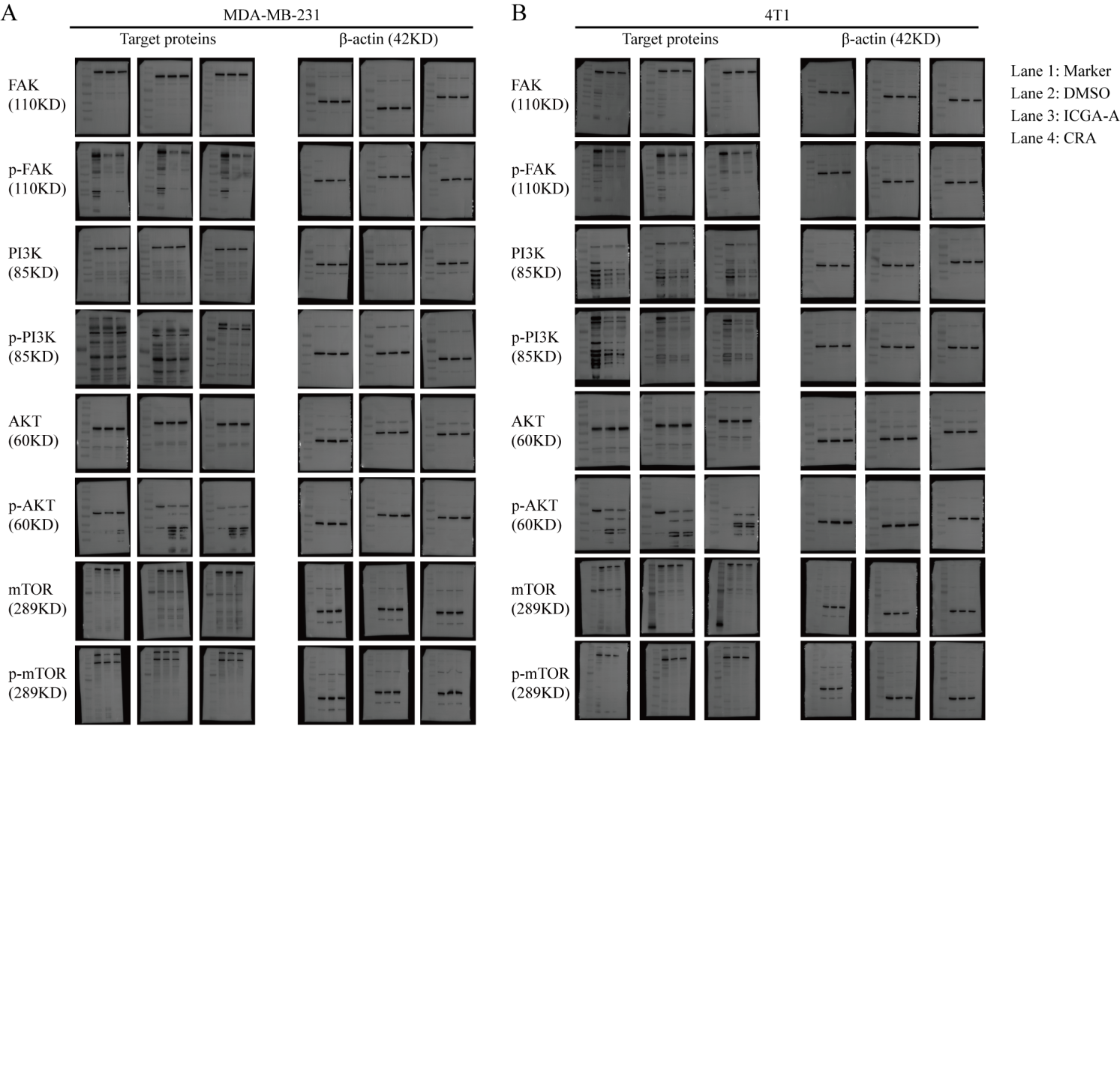


**Supplementary Figue 3: Original images of the Western blot analysis for three independent experiments.**

(A)Original Western blot images showing the total and phosphorylated levels of FAK, PI3K, AKT, and mTOR in MDA-MB-231 cells treated with ICGA-A and CRA for 48 hours.

(B)Original Western blot images showing the total and phosphorylated levels of FAK, PI3K, AKT, and mTOR in 4T1 cells treated with ICGA-A and CRA for 48 hours.

2.4 Supplementary Figue 4


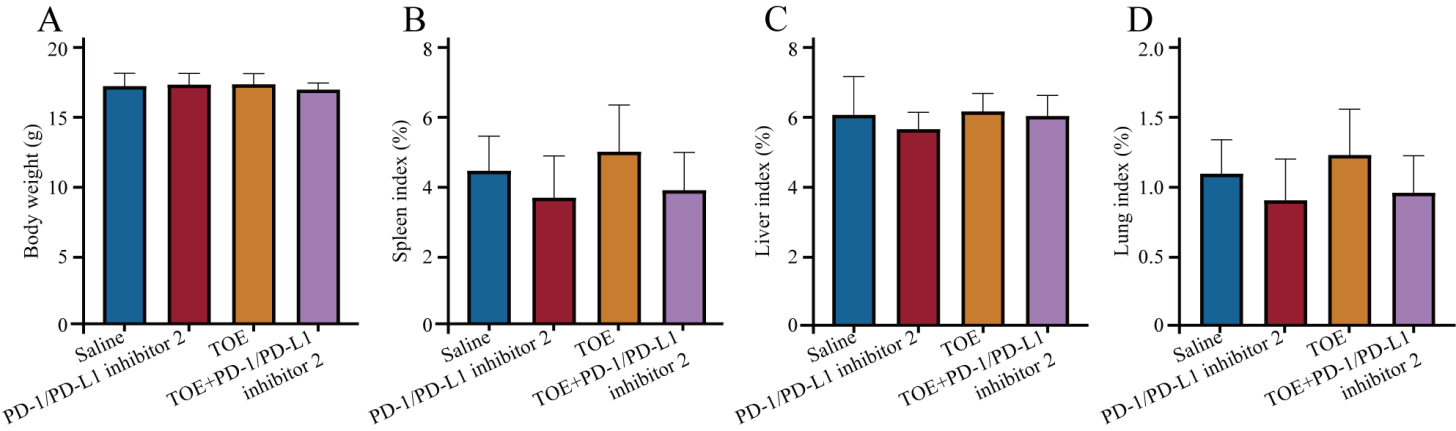


**Supplementary Figue 4: Effects of TOE combined with PD-1/PD-L1 inhibitor 2 on body weight and organ indices in breast cancer mouse models at Day 14.**

1. Body weight. (B) Spleen index. (C)Liver index. (D) Lung index. The organ index was calculated as the percentage of organ weight relative to body weight. Data are expressed as mean ± SEM. Statistical significance was determined using one-way ANOVA, with *p* < 0.05 considered statistically significant.

2.5 Supplementary Figue 5


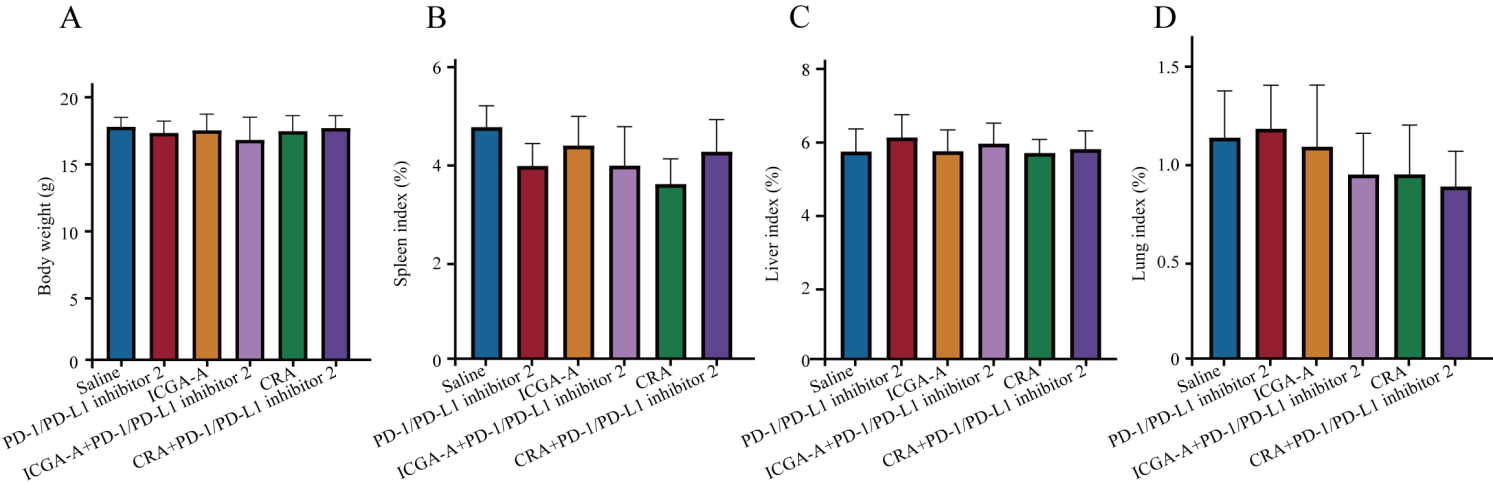


**Supplementary Figue 5: Impact of ICGA-A and CRA in combination with PD-1/PD-L1 inhibitor 2 on body weight and organ indices in a breast cancer mouse model on day 14.**

1. Body weight. (B) Spleen index. (C)Liver index. (D) Lung index. The organ index was calculated as the percentage of organ weight relative to body weight. Data are expressed as mean ± SEM. Statistical significance was determined using one-way ANOVA, with *p* < 0.05 considered statistically significant.

2.6 Supplementary Figue 6

**Supplementary Figue 6: Impact of ICGA-A and CRA in combination with PD-1/PD-L1 inhibitor 2 on Treg cells in the immune microenvironment.**

1. Representative flow cytometry plots depicting regulatory T cells (Tregs, CD3+, CD25+, Foxp3+) were obtained after different treatments.
2. The levels of Tregs were quantified by flow cytometry analysis (n = 5). Statistical significance was determined using Tukey’s multiple comparisons test. The following adjusted p-values were obtained for each comparison: Saline vs. PD-1/PD-L1 inhibitor 2: 0.9996; Saline vs. ICGA-A: 0.9996; Saline vs. ICGA-A+PD-1/PD-L1 inhibitor 2: >0.9999; Saline vs. CRA: 0.5981; Saline vs. CRA+PD-1/PD-L1 inhibitor 2: 0.9495; PD-1/PD-L1 inhibitor 2 vs. ICGA-A: 0.9896; PD-1/PD-L1 inhibitor 2 vs. ICGA-A+PD-1/PD-L1 inhibitor 2: >0.9999; PD-1/PD-L1 inhibitor 2 vs. CRA: 0.4135; PD-1/PD-L1 inhibitor 2 vs. CRA+PD-1/PD-L1 inhibitor 2: 0.8412; ICGA-A vs. ICGA-A+PD-1/PD-L1 inhibitor 2: 0.9952; ICGA-A vs. CRA: 0.7731; ICGA-A vs. CRA+PD-1/PD-L1 inhibitor 2: 0.9917; ICGA-A+PD-1/PD-L1 inhibitor 2 vs. CRA: 0.4670; ICGA-A+PD-1/PD-L1 inhibitor 2 vs. CRA+PD-1/PD-L1 inhibitor 2: 0.8813; CRA vs. CRA+PD-1/PD-L1 inhibitor 2: 0.9746.
3. The levels of M2 macrophages were quantified using flow cytometry analysis (n = 5). Statistical significance was assessed using Tukey's multiple comparisons test. The following adjusted p-values were obtained for each comparison: Saline vs. PD-1/PD-L1 inhibitor 2: >0.9999; Saline vs. ICGA-A: 0.9903; Saline vs. ICGA-A+PD-1/PD-L1 inhibitor 2: >0.9999; Saline vs. CRA: 0.9092; Saline vs. CRA+PD-1/PD-L1 inhibitor 2: 0.8967; PD-1/PD-L1 inhibitor 2 vs. ICGA-A: 0.9723; PD-1/PD-L1 inhibitor 2 vs. ICGA-A+PD-1/PD-L1 inhibitor 2: 0.9994; PD-1/PD-L1 inhibitor 2 vs. CRA: 0.8454; PD-1/PD-L1 inhibitor 2 vs. CRA+PD-1/PD-L1 inhibitor 2: 0.8289; ICGA-A vs. ICGA-A+PD-1/PD-L1 inhibitor 2: 0.9980; ICGA-A vs. CRA: 0.9982; ICGA-A vs. CRA+PD-1/PD-L1 inhibitor 2: 0.9973; ICGA-A+PD-1/PD-L1 inhibitor 2 vs. CRA: 0.9568; ICGA-A+PD-1/PD-L1 inhibitor 2 vs. CRA+PD-1/PD-L1 inhibitor 2: 0.9489; CRA vs. CRA+PD-1/PD-L1 inhibitor 2: >0.9999.
